# 2-Dibutyl­amino-1-(2,7-dichloro-9*H*-fluoren-4-yl)ethanol

**DOI:** 10.1107/S1600536810037566

**Published:** 2010-09-25

**Authors:** Hoong-Kun Fun, Chin Sing Yeap, A. M. Vijesh, Arun M Isloor, P. K. Vasudeva

**Affiliations:** a X-ray Crystallography Unit, School of Physics, Universiti Sains Malaysia, 11800 USM, Penang, Malaysia; b SeQuent Scientific Ltd, No. 120 A & B, Industrial Area, Baikampady, New Mangalore, Karnataka, 575 011, India; c Organic Chemistry Division, Department of Chemistry, National Institute of Technology-Karnataka, Surathkal, Mangalore 575 025, India

## Abstract

In the title compound, C_23_H_29_Cl_2_NO, the fluorene ring is essentially planar, with a maximum deviation from the mean plane of 0.041 (1) Å. The amine group adopts a pyramidal configuration, the sum of the bond angles being 336.2 (3)°. In the crystal, the mol­ecules are linked into dimers by inter­molecular O—H⋯N and C—H⋯O hydrogen bonds. Weak C—H⋯π and π–π [centroid–centroid distance = 3.7544 (7) Å] inter­actions are also observed.

## Related literature

For general background and applications of fluorene derivatives, see: Reinhardt *et al.* (1998 ▶); Yao & Belfield (2005 ▶); Werts *et al.* (2004 ▶); Belfield *et al.* (2009 ▶); Sun *et al.* (2009 ▶); Park *et al.* (2009 ▶); Kotaka *et al.* (2010 ▶); Wong *et al.* (2005 ▶); Beulter *et al.* (2007 ▶). For the stability of the temperature controller used in the data collection, see: Cosier & Glazer (1986 ▶).
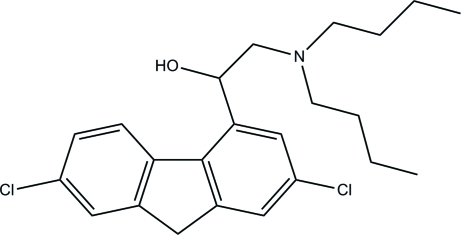


## Experimental

### 

#### Crystal data

C_23_H_29_Cl_2_NO
*M* *_r_* = 406.37
Triclinic, 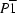

*a* = 10.0009 (4) Å
*b* = 10.8847 (4) Å
*c* = 11.0853 (4) Å
α = 68.161 (1)°
β = 70.999 (1)°
γ = 88.904 (1)°
*V* = 1051.90 (7) Å^3^
*Z* = 2
Mo *K*α radiation
μ = 0.32 mm^−1^
*T* = 100 K
0.34 × 0.28 × 0.20 mm

#### Data collection

Bruker APEXII DUO CCD area-detector diffractometer
Absorption correction: multi-scan (*SADABS*; Bruker, 2009 ▶) *T*_min_ = 0.900, *T*_max_ = 0.937
19180 measured reflections
6052 independent reflections
5448 reflections with *I* > 2σ(*I*)
*R*_int_ = 0.025

#### Refinement

*R*[*F* ^2^ > 2σ(*F* ^2^)] = 0.035
*wR*(*F* ^2^) = 0.135
*S* = 1.15
6052 reflections
250 parameters
H atoms treated by a mixture of independent and constrained refinement
Δρ_max_ = 0.65 e Å^−3^
Δρ_min_ = −0.39 e Å^−3^

### 

Data collection: *APEX2* (Bruker, 2009 ▶); cell refinement: *SAINT* (Bruker, 2009 ▶); data reduction: *SAINT*; program(s) used to solve structure: *SHELXTL* (Sheldrick, 2008 ▶); program(s) used to refine structure: *SHELXTL*; molecular graphics: *SHELXTL*; software used to prepare material for publication: *SHELXTL* and *PLATON* (Spek, 2009 ▶).

## Supplementary Material

Crystal structure: contains datablocks global, I. DOI: 10.1107/S1600536810037566/fj2335sup1.cif

Structure factors: contains datablocks I. DOI: 10.1107/S1600536810037566/fj2335Isup2.hkl

Additional supplementary materials:  crystallographic information; 3D view; checkCIF report

## Figures and Tables

**Table 1:** Hydrogen-bond geometry (Å, °) *Cg*2 and *Cg*3 are the centroids of the C2–C7 and C1/C9–C13 benzene rings, respectively.

*D*—H⋯*A*	*D*—H	H⋯*A*	*D*⋯*A*	*D*—H⋯*A*
O1—H1*O*1⋯N1^i^	0.89 (2)	1.99 (2)	2.8762 (13)	175 (2)
C14—H14*A*⋯O1^i^	0.98	2.57	3.1831 (15)	121
C8—H8*A*⋯*Cg*2^ii^	0.97	2.98	3.6293 (13)	126
C22—H22*A*⋯*Cg*2^iii^	0.97	2.82	3.6730 (15)	148
C23—H23*B*⋯*Cg*3^i^	0.96	2.92	3.7920 (18)	152

Symmetry codes: (i) 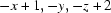; (ii) 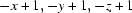; (iii) 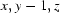.

**Fig. 1.**
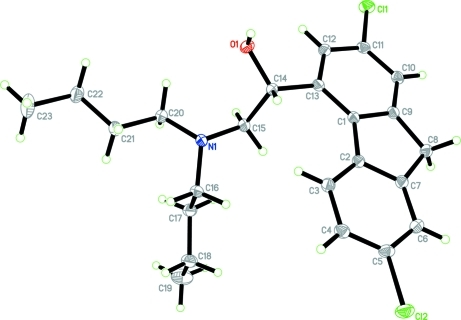
The title compound with atom labels and 50% probability displacement ellipsoids for non-H atoms.

**Fig. 2.**
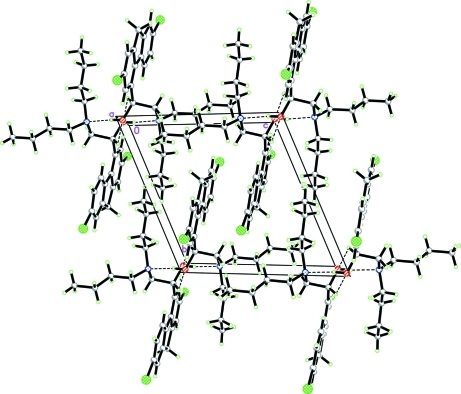
The crystal packing of title compound, viewed down the a axis showing the molecules link into dimers.

## supplementary crystallographic information

### Comment

The fluorene ring is a π-conjugated system that enables facile synthetic manipulation. The synthesis and characterization of fluorene derivatives have attracted much attention in recent years because of their interesting properties and potential applications in various fields, such as two-photon absorption dyes, fluorescent probes, and light-emitting materials (Reinhardt *et al.*, 1998; Yao & Belfield, 2005; Werts *et al.*, 2004; Belfield *et al.*, 2009; Sun *et al.*, 2009; Park *et al.*, 2009; Kotaka *et al.*, 2010; Wong et *al.*, 2005). Fluorene derivatives are also possess biological activities like antimalerial property (Beulter *et al.*, 2007). These diverse applications of fluorene derivatives led us to explore the crystal structure of the title compound.

In the title compound (Fig. 1), the fluorene ring is essentially coplanar with maximum deviation of 0.041 (1) Å at atom C5. The amine group adopts a pyramidal configuration. In the crystal structure, the molecules are linked into dimers (Fig. 2) by intermolecular O1—H1O1···N1 and C14—H14A···O1 hydrogen bonds (Table 1). Weak C—H···π and π···π interactions are observed. *Cg*1···*Cg*3^ii^ of 3.7544 (7) Å. *Cg*1 is centroid of benzene ring C1–C2/C7–C9.

### Experimental

The title compound was recrystallized from acetone. *M.p*. 352–354 K. The sample was a gift from SeQuent Scientific Ltd., No: 120 A & B, Industrial Area, Baikampady, New Mangalore, Karnataka, 575 011, India.

### Refinement

The O-bound hydrogen atom was located from difference Fourier map and refined freely. The rest of the hydrogen atoms were positioned geometrically and refined using a riding model. A rotating-group model was applied for the methyl groups.

### Crystal data

**Table:**

C_23_H_29_Cl_2_NO	*Z* = 2
*M**_r_* = 406.37	*F*(000) = 432
Triclinic, *P*1	*D*_x_ = 1.283 Mg m^−^^3^
Hall symbol: -P 1	Mo *K*α radiation, λ = 0.71073 Å
*a* = 10.0009 (4) Å	Cell parameters from 9903 reflections
*b* = 10.8847 (4) Å	θ = 2.8–35.1°
*c* = 11.0853 (4) Å	µ = 0.32 mm^−^^1^
α = 68.161 (1)°	*T* = 100 K
β = 70.999 (1)°	Block, colourless
γ = 88.904 (1)°	0.34 × 0.28 × 0.20 mm
*V* = 1051.90 (7) Å^3^	

### Data collection

**Table:**

Bruker APEXII DUO CCD area-detector diffractometer	6052 independent reflections
Radiation source: fine-focus sealed tube	5448 reflections with *I* > 2σ(*I*)
graphite	*R*_int_ = 0.025
φ and ω scans	θ_max_ = 30.0°, θ_min_ = 2.0°
Absorption correction: multi-scan (*SADABS*; Bruker, 2009)	*h* = −14→14
*T*_min_ = 0.900, *T*_max_ = 0.937	*k* = −13→15
19180 measured reflections	*l* = −15→15

### Refinement

**Table:**

Refinement on *F*^2^	Primary atom site location: structure-invariant direct methods
Least-squares matrix: full	Secondary atom site location: difference Fourier map
*R*[*F*^2^ > 2σ(*F*^2^)] = 0.035	Hydrogen site location: inferred from neighbouring sites
*wR*(*F*^2^) = 0.135	H atoms treated by a mixture of independent and constrained refinement
*S* = 1.15	*w* = 1/[σ^2^(*F*_o_^2^) + (0.0878*P*)^2^ + 0.1785*P*] where *P* = (*F*_o_^2^ + 2*F*_c_^2^)/3
6052 reflections	(Δ/σ)_max_ = 0.001
250 parameters	Δρ_max_ = 0.65 e Å^−^^3^
0 restraints	Δρ_min_ = −0.39 e Å^−^^3^

### Special details

**Table:**

Experimental. The crystal was placed in the cold stream of an Oxford Cryosystems Cobra open-flow nitrogen cryostat (Cosier & Glazer, 1986) operating at 100.0 (1) K.
Geometry. All e.s.d.'s (except the e.s.d. in the dihedral angle between two l.s. planes) are estimated using the full covariance matrix. The cell e.s.d.'s are taken into account individually in the estimation of e.s.d.'s in distances, angles and torsion angles; correlations between e.s.d.'s in cell parameters are only used when they are defined by crystal symmetry. An approximate (isotropic) treatment of cell e.s.d.'s is used for estimating e.s.d.'s involving l.s. planes.
Refinement. Refinement of *F*^2^ against ALL reflections. The weighted *R*-factor *wR* and goodness of fit *S* are based on *F*^2^, conventional *R*-factors *R* are based on *F*, with *F* set to zero for negative *F*^2^. The threshold expression of *F*^2^ > σ(*F*^2^) is used only for calculating *R*-factors(gt) *etc*. and is not relevant to the choice of reflections for refinement. *R*-factors based on *F*^2^ are statistically about twice as large as those based on *F*, and *R*- factors based on ALL data will be even larger.

### Fractional atomic coordinates and isotropic or equivalent isotropic displacement parameters (Å^2^)

**Table:**

	*x*	*y*	*z*	*U*_iso_*/*U*_eq_	
Cl1	−0.09361 (3)	0.21897 (3)	0.88976 (3)	0.02143 (10)	
Cl2	0.91082 (3)	0.72785 (3)	0.47680 (3)	0.02310 (10)	
O1	0.34512 (9)	−0.00277 (8)	0.98844 (9)	0.01700 (17)	
N1	0.61607 (10)	0.00676 (9)	0.76424 (10)	0.01474 (18)	
C1	0.37158 (11)	0.35655 (11)	0.76122 (11)	0.01307 (19)	
C2	0.51590 (11)	0.42961 (11)	0.70234 (11)	0.0137 (2)	
C3	0.64840 (12)	0.38878 (11)	0.70641 (12)	0.0157 (2)	
H3A	0.6557	0.3002	0.7557	0.019*	
C4	0.76946 (12)	0.48193 (12)	0.63599 (12)	0.0174 (2)	
H4A	0.8580	0.4555	0.6380	0.021*	
C5	0.75782 (12)	0.61409 (12)	0.56297 (12)	0.0170 (2)	
C6	0.62725 (12)	0.65831 (11)	0.55809 (11)	0.0168 (2)	
H6A	0.6208	0.7472	0.5093	0.020*	
C7	0.50725 (12)	0.56512 (11)	0.62855 (11)	0.0147 (2)	
C8	0.35494 (12)	0.58751 (11)	0.64065 (12)	0.0166 (2)	
H8A	0.3211	0.6472	0.6879	0.020*	
H8B	0.3446	0.6239	0.5503	0.020*	
C9	0.27675 (11)	0.44936 (11)	0.72460 (11)	0.0143 (2)	
C10	0.13297 (12)	0.41040 (12)	0.76341 (11)	0.0161 (2)	
H10A	0.0709	0.4719	0.7390	0.019*	
C11	0.08464 (11)	0.27561 (12)	0.84032 (12)	0.0160 (2)	
C12	0.17583 (11)	0.18198 (11)	0.87841 (11)	0.0154 (2)	
H12A	0.1399	0.0928	0.9301	0.019*	
C13	0.32054 (11)	0.22131 (11)	0.83943 (11)	0.01356 (19)	
C14	0.41897 (11)	0.11665 (11)	0.87700 (11)	0.0141 (2)	
H14A	0.4898	0.1530	0.9023	0.017*	
C15	0.49599 (12)	0.08479 (11)	0.74948 (12)	0.0154 (2)	
H15A	0.4284	0.0352	0.7336	0.018*	
H15B	0.5313	0.1676	0.6691	0.018*	
C16	0.74550 (12)	0.06358 (12)	0.63995 (12)	0.0162 (2)	
H16A	0.8249	0.0192	0.6611	0.019*	
H16B	0.7652	0.1569	0.6218	0.019*	
C17	0.74054 (12)	0.05380 (12)	0.50737 (12)	0.0188 (2)	
H17A	0.7301	−0.0393	0.5209	0.023*	
H17B	0.6582	0.0929	0.4878	0.023*	
C18	0.87478 (13)	0.12510 (13)	0.38423 (12)	0.0220 (2)	
H18A	0.8838	0.2184	0.3702	0.026*	
H18B	0.9570	0.0873	0.4053	0.026*	
C19	0.87480 (18)	0.11485 (18)	0.25151 (15)	0.0351 (3)	
H19A	0.9637	0.1568	0.1791	0.053*	
H19B	0.7982	0.1585	0.2259	0.053*	
H19C	0.8625	0.0227	0.2655	0.053*	
C20	0.57620 (12)	−0.13645 (11)	0.80309 (12)	0.0167 (2)	
H20A	0.5521	−0.1476	0.7295	0.020*	
H20B	0.4922	−0.1680	0.8859	0.020*	
C21	0.69500 (13)	−0.22106 (12)	0.82942 (12)	0.0184 (2)	
H21A	0.7679	−0.2082	0.7415	0.022*	
H21B	0.7381	−0.1912	0.8820	0.022*	
C22	0.64153 (15)	−0.36850 (13)	0.90795 (13)	0.0229 (2)	
H22A	0.5982	−0.3982	0.8554	0.027*	
H22B	0.5687	−0.3813	0.9959	0.027*	
C23	0.7601 (2)	−0.45345 (16)	0.93422 (16)	0.0342 (3)	
H23A	0.7230	−0.5460	0.9768	0.051*	
H23B	0.7965	−0.4309	0.9944	0.051*	
H23C	0.8353	−0.4369	0.8480	0.051*	
H1O1	0.359 (2)	0.001 (2)	1.062 (2)	0.036 (5)*	

### Atomic displacement parameters (Å^2^)

**Table:**

	*U*^11^	*U*^22^	*U*^33^	*U*^12^	*U*^13^	*U*^23^
Cl1	0.01152 (14)	0.02463 (17)	0.02342 (16)	0.00024 (10)	−0.00496 (11)	−0.00501 (12)
Cl2	0.01794 (15)	0.02340 (17)	0.02422 (16)	−0.00603 (11)	−0.00230 (11)	−0.00900 (12)
O1	0.0163 (4)	0.0142 (4)	0.0161 (4)	−0.0002 (3)	−0.0040 (3)	−0.0025 (3)
N1	0.0124 (4)	0.0129 (4)	0.0171 (4)	0.0023 (3)	−0.0027 (3)	−0.0058 (3)
C1	0.0123 (4)	0.0138 (5)	0.0132 (4)	0.0025 (3)	−0.0043 (4)	−0.0055 (4)
C2	0.0134 (5)	0.0145 (5)	0.0139 (4)	0.0019 (4)	−0.0047 (4)	−0.0063 (4)
C3	0.0145 (5)	0.0156 (5)	0.0176 (5)	0.0020 (4)	−0.0061 (4)	−0.0065 (4)
C4	0.0135 (5)	0.0205 (5)	0.0195 (5)	0.0011 (4)	−0.0053 (4)	−0.0095 (4)
C5	0.0152 (5)	0.0194 (5)	0.0157 (5)	−0.0027 (4)	−0.0029 (4)	−0.0078 (4)
C6	0.0184 (5)	0.0150 (5)	0.0161 (5)	0.0000 (4)	−0.0056 (4)	−0.0052 (4)
C7	0.0156 (5)	0.0146 (5)	0.0141 (5)	0.0020 (4)	−0.0048 (4)	−0.0060 (4)
C8	0.0158 (5)	0.0141 (5)	0.0184 (5)	0.0031 (4)	−0.0061 (4)	−0.0045 (4)
C9	0.0139 (5)	0.0147 (5)	0.0145 (5)	0.0034 (4)	−0.0048 (4)	−0.0060 (4)
C10	0.0136 (5)	0.0177 (5)	0.0167 (5)	0.0050 (4)	−0.0057 (4)	−0.0061 (4)
C11	0.0115 (4)	0.0183 (5)	0.0166 (5)	0.0012 (4)	−0.0036 (4)	−0.0061 (4)
C12	0.0126 (5)	0.0153 (5)	0.0162 (5)	0.0016 (4)	−0.0038 (4)	−0.0048 (4)
C13	0.0125 (4)	0.0140 (5)	0.0135 (4)	0.0029 (4)	−0.0038 (4)	−0.0053 (4)
C14	0.0126 (4)	0.0118 (5)	0.0150 (5)	0.0014 (3)	−0.0033 (4)	−0.0034 (4)
C15	0.0138 (5)	0.0147 (5)	0.0173 (5)	0.0033 (4)	−0.0050 (4)	−0.0061 (4)
C16	0.0126 (5)	0.0163 (5)	0.0174 (5)	0.0007 (4)	−0.0021 (4)	−0.0066 (4)
C17	0.0180 (5)	0.0181 (5)	0.0176 (5)	0.0001 (4)	−0.0025 (4)	−0.0070 (4)
C18	0.0179 (5)	0.0252 (6)	0.0183 (5)	0.0014 (4)	−0.0027 (4)	−0.0064 (4)
C19	0.0343 (8)	0.0468 (9)	0.0195 (6)	−0.0015 (6)	−0.0029 (5)	−0.0130 (6)
C20	0.0168 (5)	0.0130 (5)	0.0183 (5)	0.0016 (4)	−0.0042 (4)	−0.0057 (4)
C21	0.0204 (5)	0.0158 (5)	0.0180 (5)	0.0054 (4)	−0.0047 (4)	−0.0073 (4)
C22	0.0339 (7)	0.0158 (5)	0.0187 (5)	0.0064 (5)	−0.0087 (5)	−0.0069 (4)
C23	0.0539 (9)	0.0255 (7)	0.0317 (7)	0.0212 (6)	−0.0222 (7)	−0.0146 (6)

### Geometric parameters (Å, °)

**Table:**

Cl1—C11	1.7358 (11)	C13—C14	1.5202 (15)
Cl2—C5	1.7400 (12)	C14—C15	1.5354 (15)
O1—C14	1.4175 (13)	C14—H14A	0.9800
O1—H1O1	0.89 (2)	C15—H15A	0.9700
N1—C15	1.4751 (14)	C15—H15B	0.9700
N1—C20	1.4788 (14)	C16—C17	1.5290 (16)
N1—C16	1.4805 (14)	C16—H16A	0.9700
C1—C13	1.4037 (15)	C16—H16B	0.9700
C1—C9	1.4119 (14)	C17—C18	1.5252 (16)
C1—C2	1.4785 (15)	C17—H17A	0.9700
C2—C3	1.3989 (15)	C17—H17B	0.9700
C2—C7	1.4127 (15)	C18—C19	1.5162 (19)
C3—C4	1.3950 (16)	C18—H18A	0.9700
C3—H3A	0.9300	C18—H18B	0.9700
C4—C5	1.3881 (17)	C19—H19A	0.9600
C4—H4A	0.9300	C19—H19B	0.9600
C5—C6	1.3936 (16)	C19—H19C	0.9600
C6—C7	1.3878 (15)	C20—C21	1.5261 (16)
C6—H6A	0.9300	C20—H20A	0.9700
C7—C8	1.5082 (15)	C20—H20B	0.9700
C8—C9	1.5062 (16)	C21—C22	1.5197 (17)
C8—H8A	0.9700	C21—H21A	0.9700
C8—H8B	0.9700	C21—H21B	0.9700
C9—C10	1.3847 (15)	C22—C23	1.5259 (19)
C10—C11	1.3932 (16)	C22—H22A	0.9700
C10—H10A	0.9300	C22—H22B	0.9700
C11—C12	1.3941 (15)	C23—H23A	0.9600
C12—C13	1.3940 (15)	C23—H23B	0.9600
C12—H12A	0.9300	C23—H23C	0.9600
			
C14—O1—H1O1	105.1 (14)	N1—C15—H15A	109.0
C15—N1—C20	111.58 (9)	C14—C15—H15A	109.0
C15—N1—C16	111.49 (9)	N1—C15—H15B	109.0
C20—N1—C16	113.09 (9)	C14—C15—H15B	109.0
C13—C1—C9	119.98 (10)	H15A—C15—H15B	107.8
C13—C1—C2	132.12 (10)	N1—C16—C17	116.53 (9)
C9—C1—C2	107.90 (9)	N1—C16—H16A	108.2
C3—C2—C7	119.17 (10)	C17—C16—H16A	108.2
C3—C2—C1	132.54 (10)	N1—C16—H16B	108.2
C7—C2—C1	108.28 (9)	C17—C16—H16B	108.2
C4—C3—C2	119.50 (11)	H16A—C16—H16B	107.3
C4—C3—H3A	120.2	C18—C17—C16	111.72 (10)
C2—C3—H3A	120.2	C18—C17—H17A	109.3
C5—C4—C3	119.99 (10)	C16—C17—H17A	109.3
C5—C4—H4A	120.0	C18—C17—H17B	109.3
C3—C4—H4A	120.0	C16—C17—H17B	109.3
C4—C5—C6	121.95 (10)	H17A—C17—H17B	107.9
C4—C5—Cl2	118.82 (9)	C19—C18—C17	113.12 (11)
C6—C5—Cl2	119.23 (9)	C19—C18—H18A	109.0
C7—C6—C5	117.74 (10)	C17—C18—H18A	109.0
C7—C6—H6A	121.1	C19—C18—H18B	109.0
C5—C6—H6A	121.1	C17—C18—H18B	109.0
C6—C7—C2	121.64 (10)	H18A—C18—H18B	107.8
C6—C7—C8	128.05 (10)	C18—C19—H19A	109.5
C2—C7—C8	110.31 (10)	C18—C19—H19B	109.5
C9—C8—C7	102.80 (9)	H19A—C19—H19B	109.5
C9—C8—H8A	111.2	C18—C19—H19C	109.5
C7—C8—H8A	111.2	H19A—C19—H19C	109.5
C9—C8—H8B	111.2	H19B—C19—H19C	109.5
C7—C8—H8B	111.2	N1—C20—C21	112.70 (9)
H8A—C8—H8B	109.1	N1—C20—H20A	109.1
C10—C9—C1	121.57 (10)	C21—C20—H20A	109.1
C10—C9—C8	127.77 (10)	N1—C20—H20B	109.1
C1—C9—C8	110.66 (9)	C21—C20—H20B	109.1
C9—C10—C11	117.54 (10)	H20A—C20—H20B	107.8
C9—C10—H10A	121.2	C22—C21—C20	112.38 (10)
C11—C10—H10A	121.2	C22—C21—H21A	109.1
C10—C11—C12	122.07 (10)	C20—C21—H21A	109.1
C10—C11—Cl1	120.11 (9)	C22—C21—H21B	109.1
C12—C11—Cl1	117.81 (9)	C20—C21—H21B	109.1
C13—C12—C11	120.35 (10)	H21A—C21—H21B	107.9
C13—C12—H12A	119.8	C21—C22—C23	112.52 (12)
C11—C12—H12A	119.8	C21—C22—H22A	109.1
C12—C13—C1	118.49 (10)	C23—C22—H22A	109.1
C12—C13—C14	119.41 (10)	C21—C22—H22B	109.1
C1—C13—C14	122.04 (9)	C23—C22—H22B	109.1
O1—C14—C13	112.68 (9)	H22A—C22—H22B	107.8
O1—C14—C15	108.89 (9)	C22—C23—H23A	109.5
C13—C14—C15	108.43 (9)	C22—C23—H23B	109.5
O1—C14—H14A	108.9	H23A—C23—H23B	109.5
C13—C14—H14A	108.9	C22—C23—H23C	109.5
C15—C14—H14A	108.9	H23A—C23—H23C	109.5
N1—C15—C14	112.91 (9)	H23B—C23—H23C	109.5
			
C13—C1—C2—C3	−0.9 (2)	C9—C10—C11—C12	0.31 (17)
C9—C1—C2—C3	179.75 (11)	C9—C10—C11—Cl1	−178.74 (8)
C13—C1—C2—C7	178.18 (11)	C10—C11—C12—C13	−0.25 (18)
C9—C1—C2—C7	−1.15 (12)	Cl1—C11—C12—C13	178.83 (9)
C7—C2—C3—C4	−0.98 (16)	C11—C12—C13—C1	−0.12 (17)
C1—C2—C3—C4	178.04 (11)	C11—C12—C13—C14	−177.47 (10)
C2—C3—C4—C5	0.21 (17)	C9—C1—C13—C12	0.40 (16)
C3—C4—C5—C6	0.51 (17)	C2—C1—C13—C12	−178.86 (11)
C3—C4—C5—Cl2	179.76 (9)	C9—C1—C13—C14	177.68 (10)
C4—C5—C6—C7	−0.40 (17)	C2—C1—C13—C14	−1.58 (18)
Cl2—C5—C6—C7	−179.66 (8)	C12—C13—C14—O1	−20.91 (14)
C5—C6—C7—C2	−0.40 (16)	C1—C13—C14—O1	161.83 (10)
C5—C6—C7—C8	179.25 (10)	C12—C13—C14—C15	99.69 (11)
C3—C2—C7—C6	1.10 (16)	C1—C13—C14—C15	−77.56 (13)
C1—C2—C7—C6	−178.14 (10)	C20—N1—C15—C14	99.92 (11)
C3—C2—C7—C8	−178.62 (10)	C16—N1—C15—C14	−132.56 (10)
C1—C2—C7—C8	2.15 (12)	O1—C14—C15—N1	−69.47 (11)
C6—C7—C8—C9	178.09 (11)	C13—C14—C15—N1	167.61 (9)
C2—C7—C8—C9	−2.22 (12)	C15—N1—C16—C17	−68.12 (12)
C13—C1—C9—C10	−0.34 (16)	C20—N1—C16—C17	58.58 (13)
C2—C1—C9—C10	179.08 (10)	N1—C16—C17—C18	175.50 (10)
C13—C1—C9—C8	−179.72 (10)	C16—C17—C18—C19	178.77 (12)
C2—C1—C9—C8	−0.29 (12)	C15—N1—C20—C21	−175.79 (9)
C7—C8—C9—C10	−177.83 (11)	C16—N1—C20—C21	57.56 (12)
C7—C8—C9—C1	1.49 (12)	N1—C20—C21—C22	163.47 (10)
C1—C9—C10—C11	−0.01 (17)	C20—C21—C22—C23	179.89 (11)
C8—C9—C10—C11	179.25 (11)		

### Hydrogen-bond geometry (Å, °)

**Table:**

Cg2 and Cg3 are the centroids of the C2–C7 and C1/C9–C13 benzene rings, respectively.

**Table:**

*D*—H···*A*	*D*—H	H···*A*	*D*···*A*	*D*—H···*A*
O1—H1O1···N1^i^	0.89 (2)	1.99 (2)	2.8762 (13)	175 (2)
C14—H14A···O1^i^	0.98	2.57	3.1831 (15)	121
C8—H8A···Cg2^ii^	0.97	2.98	3.6293 (13)	126
C22—H22A···Cg2^iii^	0.97	2.82	3.6730 (15)	148
C23—H23B···Cg3^i^	0.96	2.92	3.7920 (18)	152

Symmetry codes: (i) −*x*+1, −*y*, −*z*+2; (ii) −*x*+1, −*y*+1, −*z*+1; (iii) *x*, *y*−1, *z*.
